# Early Stages of Age-Related Macular Degeneration: Racial/Ethnic Differences and Proposal of a New Classification Incorporating Emerging Concept of Choroidal Pathology

**DOI:** 10.3390/jcm11216274

**Published:** 2022-10-25

**Authors:** Mariko Sasaki, Ryo Kawasaki, Yasuo Yanagi

**Affiliations:** 1 Department of Ophthalmology, Keio University School of Medicine, 35 Shinanomachi, Shinjuku-ku, Tokyo 160-8582, Japan; 2 Department of Ophthalmology, Tokyo Medical Center, Tokyo 152-8902, Japan; 3 Department of Vision Informatics (Topcon), Osaka University Graduate School of Medicine, Osaka 565-0871, Japan; 4 Department of Ophthalmology and Micro-Technology, Yokohama City University, Kanazawa 236-0027, Japan; 5 Singapore Eye Research Institute, Singapore National Eye Centre, Singapore 169608, Singapore

**Keywords:** age-related macular degeneration (AMD), early AMD, macular neovascularization (MNV), drusen, pigmentary abnormalities, pachychoroid, multimodal imaging, optical coherence tomography (OCT), classification, ethnicity

## Abstract

The progression of age-related macular degeneration (AMD) is determined by environmental and genetic factors, and phenotypic or molecular risk factors have been investigated extensively. Interestingly, risk factor profiles for advanced AMD differ among individuals, and one of the causes of variation may be explained by their ethnic background. Recent advances in retinal imaging technology have led to the identification of previously unrecognized risk factors for advanced AMD on optical coherence tomography (OCT) and OCT angiography, which expands the concept of traditional imaging risk factors such as drusen and pigmentary abnormalities visible on color fundus photographs. This OCT imaging modality has identified novel pathognomonic changes for early AMD, including the associated photoreceptor, retinal pigment epithelium, and underlying choroidal changes. Regarding features of multimodal imaging associated with the presence or progression of geographic atrophy, there is an international expert consensus classification system; however, features associated with the progression of macular neovascularization (MNV) are still obscure. To make a consensus towards understanding features associated with the risk of MNV, this review focuses on the early stages of AMD by summarizing imaging characteristics and early signs and classifications in view of advanced multimodal imaging technology. Recent evidence suggests that neovascular AMD is not a single disease entity but a heterogeneous disease characterized by MNV. Besides drusen, OCT features associated with pigment abnormalities, such as shallow irregular RPE elevation (SIRE, also known as double-layer sign), pachychoroid pigment epitheliopathy, and choriocapillaris ischemia, seem to confer a high risk of MNV developing, especially for Asian populations.

## 1. Introduction

Age-related macular degeneration (AMD) is a leading cause of vision loss in elderly people worldwide [1,2], with an estimate that 288 million people will be affected by 2040 worldwide [3]. Accordingly, AMD is becoming an important healthcare problem in our aging world. 

With the development of multimodal imaging technology in recent decades, particularly optical coherence tomography (OCT) and OCT angiography (OCTA), understanding of the pathology and concept of AMD has rapidly advanced, especially on the involvement of choroidal pathology. For example, pachychoroid spectrum disease (PSD) has been recognized based on choroidal features as a distinct entity from AMD [4,5,6], and the concept of venous overload choroidopathy has subsequently been proposed, which is linked to venous decompensation in the choroid [7]. Pachydrusen have been recognized as an early sign of AMD in which drusenoid deposits form in conjunction with pachychoroid [8].

Definitions and classifications of diseases reflect contemporaneous disease concepts and imaging capabilities. Based on knowledge obtained from OCT images, an international expert consensus was reached on terms and descriptions of atrophy, including components such as outer retinal atrophy, complete retinal pigment epithelium (RPE), and outer retinal atrophy (cRORA), as well as on risk factors for atrophy development [9]. For macular neovascularization (MNV), a few new classification systems have been proposed [5,10,11]. However, consensus is still lacking on features associated with MNV progression.

Although the conventional classification system lacks the concept of choroidal pathology, a population-based study demonstrated that choroidal thickness is associated with the pathology of intermediate AMD in Asians [12]. Asian patients with AMD are more likely than White patients to have pachydrusen, and greater choroidal thickness was associated with pachydrusen [13]. A few studies in Asia have demonstrated an association between PCV and pigment abnormalities (even when typical signs of drusen are absent) [14,15], although the early stages of AMD are defined based on the presence of drusen in the White population [16,17,18,19]. These findings suggest early involvement of the choroid in the pathogenesis of AMD in Asians.

This review focuses on the early stages of AMD and summarizes characteristic early signs and classifications of AMD in view of the advances in the concept of AMD with multimodal imaging technology. Additionally, we proposed a new classification incorporating emerging concept of choroidal pathology.

## 2. Conventional AMD Classification Based on Drusen and Pigmentary Abnormalities

The Wisconsin Age-Related Maculopathy Grading System (WARMGS) [20] is a historical system that uses a macular grid for location as well as standardized circles for size and area to semi-quantitatively grade AMD lesions on stereoscopic color fundus photographs (e.g., drusen, pigment abnormalities, late AMD). In the grading system, drusen are classified based on size (<63 μm, hard drusen; ≥63 and <125 μm, hard distinct or soft drusen; ≥125 μm, soft drusen) and area, and they are characterized by type (distinct or indistinct for each druse size as well as reticular or faded) and confluence. Other characteristics of AMD are estimated in the same way (e.g., RPE degeneration, increased pigment, subretinal fibrous scar, GA).

The International Classification system [21] followed the WARMGS and was developed into a system for classifying disease severity (Table 1) because both consensus on the definitions of specific lesions and a generally accepted classification system had been lacking. In this classification system, age-related maculopathy (ARM) is defined as a degenerative disorder in persons aged ≥50 years and is classified as early ARM and late ARM. Early ARM is defined as the presence of soft drusen (≥63 μm) and RPE pigmentary abnormalities (hyperpigmentation and/or hypopigmentation), while late ARM includes GA and neovascular AMD (RPE detachment, retinal hemorrhages, and/or scars). These early grading and classification systems were the basis of AMD detection, semi-quantification, and definition on color fundus photographs, which classified AMD into early and late stages, and these systems are still used in epidemiological studies decades later.

The Age-Related Eye Disease Study (AREDS) was a large clinical trial investigating the ability of high-dose vitamin and mineral supplements to prevent the development of advanced AMD. Three classification systems were derived from the AREDS: the original classification used in the AREDS study [22]; a simplified severity scale [23]; and a nine-step severity scale [24]. The original classification system was used in this trial for patient recruitment and analysis, with AMD classified in four categories as no or minimal AMD, early AMD, intermediate AMD, and advanced AMD [22]. Early AMD is defined as the presence of small drusen (<63 μm) or a few intermediate drusen (≥63 and ≤125 μm), while intermediate AMD is defined as the presence of extensive intermediate drusen or at least one large druse (>125 μm). Advanced AMD includes neovascular AMD and central GA (Table 1). Based on the results of the AREDS studies, the latter two scales estimate the risk of developing advanced AMD [23,24] (Table 1). In the simplified severity scale, risk factors, namely, large drusen and pigmentary changes, are summed across both eyes, yielding a five-step scale (0–4). The 5-year risk of developing advanced AMD is calculated based on the number of factors present: 0, 0.5%; 1, 3%; 2, 12%; 3, 25%; and 4, 50%. Meanwhile, the nine-step severity scale is a combination of a six-step drusen area scale with a five-step pigmentary abnormality scale using stereoscopic fundus photographs, and the 5-year risk of advanced AMD increases progressively from less than 1% in step 1 to about 50% in step 9 (Table 1).

Subsequently, the Beckman Initiative for Macular Research Classification Committee proposed a clinical classification based on the AREDS study results [25]. In the classification, eyes with neither visible drusen nor pigmentary abnormalities are defined as “no signs of AMD”, whereas small drusen (<63 μm), also termed drupelets, are regarded as “normal aging” with no increased risk of progression to late AMD. Early AMD is defined as the presence of medium drusen (≥63 and <125 μm) and no pigmentary abnormalities associated with at least medium drusen (AMD pigmentary abnormalities). Intermediate AMD is defined as the presence of large drusen (≥125μm) or AMD pigmentary abnormalities (Table 1). Late AMD includes neovascular AMD and GA. The 5-year risk of progression to late AMD was similar to that in the severity scales described above, ranging from a 0.5% risk for normal aging to a 50% risk for intermediate AMD. This classification is more practical than the earlier classifications in terms of determining disease severity and predicting the risk of late AMD. Therefore, it is now widely used in clinical and research settings. Notably, subretinal drusenoid deposits (SDDs) are not included in this classification.

## 3. Stratifying the Risk of Advanced AMD: Emerging Drusen Subtypes and Pigment Abnormalities

Environmental and genetic factors, as well as phenotypic and molecular risk factors, are associated with the progression of AMD [1,2]. The risk factors of advanced AMD may be mainly dependent on ethnic background [1,2]. For example, the prevalence of AMD and the prevalence of subtypes of AMD vary in different ethnic groups; for instance, regarding neovascular AMD, PCV is more prevalent in Asians than in White ethnic groups [26]. Similarly, the presence of drusen and the prevalence of drusen subtypes vary among populations; for instance, pachydrusen are more prevalent in Asians compared to White ethnic groups [13]. Pigment abnormalities have been considered as an early sign of AMD only when accompanied with drusen in White ethnic populations [16,17,18,19]. However, recent studies suggest that pigment abnormalities alone may confer a risk of developing MNV [14,15,25,27] and may be linked with geographic atrophy (GA) in Asian populations [28]. In addition to such traditional imaging risk factors using color fundus photograph (CFP), recent advances in retinal imaging technology have led to the identification of hitherto unrecognized risk factors for advanced AMD, such as drusenoid deposits on OCT and nonexudative MNV on OCTA. These developments have led to refinements in current AMD classification systems, which incorporate associated photoreceptor, RPE, and underlying choroidal changes. Regarding multimodal imaging features associated with GA and with risk of GA progression in eyes with AMD, an international expert consensus was reached on terms and descriptions, including components of outer retinal atrophy (e.g., ellipsoid zone (EZ) disruption), cRORA (e.g., RPE perturbation with associated hypo-transmission or hyper-transmission), features frequently seen in eyes with atrophy (e.g., refractile drusen), and those conferring risk of atrophy development (e.g., hyperreflective foci, drusen, and SDDs) [29]. Although some of these features (such as SDDs) are likely to confer risk of developing MNV, there is little consensus about features associated with MNV progression. 

### 3.1. Drusen

Drusen are extracellular deposits, typically located between the basement membrane of the RPE and the inner collagenous layer of Bruch’s membrane, and visible as yellowish deposits on CFP. Traditionally, clinical classification of drusen is based on their appearances, such as the size and number, and the distinctiveness of their margin on CFPs [1,2]. They were used (and are still useful) for AMD staging and for the prediction of the likelihood of disease progression when CFP was mainly used for epidemiological studies in ophthalmology. With the advent of multimodal imaging, drusen can be classified into typical drusen (such as hard and soft drusen), cuticular drusen (also known as basal laminar drusen), reticular pseudodrusen (or SDDs), and pachydrusen [5,8]. One phenotype considered as an end-stage of soft drusen is calcification, ophthalmologically discernible as refractile drusen.

There are also several important differences between these types of drusen. First, the retinal distribution of drusen is considerably different. Soft drusen are predilected for the central macula but may be present around the macular periphery. Cuticular drusen are more widely scattered on the fundus, with symmetrical distribution patterns in both eyes. SDDs are similarly scattered on the fundus and show symmetrical distribution. Pachydrusen are also widely scattered on the fundus but may be present in a small group and with less symmetrical distribution patterns in both eyes. Additionally, pachydrusen are sometimes co-existent with soft drusen. Second, drusen contain lipid, mineral, and protein, but the composition of each druse is highly heterogeneous histologically. Finally, their changes over time may be different, and their impact on disease progression is different. 

#### 3.1.1. Soft Drusen

**Figure 1:**
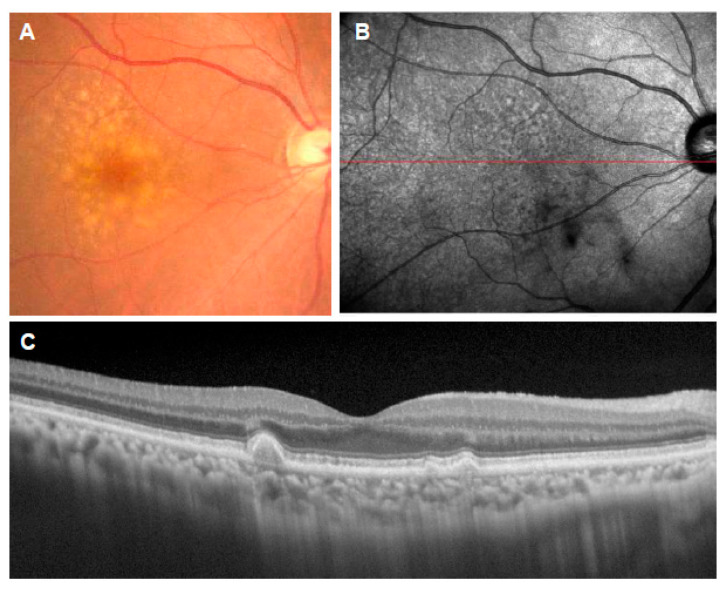
Soft drusen. (**A**): Color fundus photograph showing multiple drusen of various sizes in the macula; (**B**): near-infrared reflectance image showing the location of the OCT scan (red line); (**C**): corresponding OCT scan showing the deposition of materials under the RPE.

Soft drusen are a well-established hallmark of AMD and considered to confer an increased risk of developing both GA (or cRORA) and MNV (Figure 1). The landmark Beaver Dam study gave the first solid evidence to support that the presence of large drusen and pigmentary changes of the RPE were the predictors for the development of late AMD [30].

Histologically, soft drusen are a basal linear deposit. Extensive histological analysis disclosed that a major component is large apolipoprotein-derived lipids containing lipoproteins B, E, and cholesterol, which are presumably secreted by the RPE. Soft drusen are considered to form when secretions of functional RPE accumulate in the sub-RPE space due to an impairment of efflux across aged Bruch’s membrane. Lipids also include esterified and unesterified cholesterol. Lipid accumulation is considered as a major age-related change involved in initiating and sustaining soft drusen in AMD [30].

Recent retinal imaging studies provided newer insights into the retino-choroidal anatomical changes associated with soft drusen. Soft drusen initially grow slowly and increase in volume and then abruptly collapse, leaving atrophy. Anterior migration of RPE, especially atop drusen, occurs during this process [31]. In a two-year follow-up study, increased drusen volume was associated with the progression to cRORA but may be less associated with MNV [32]. 

Interestingly, compared with control eyes without large drusen or SDDs, the central maculas of eyes at lower risk with unilateral large drusen were thicker, whereas higher-risk eyes with SDDs were thinner in cross-sectional multivariate analyses, suggesting that macular thickness in intermediate AMD varies with disease severity and that there were small increases in eyes with large drusen and decreases in eyes with SDDs. The investigators assumed that active processes possibly related to neuroinflammation and neurodegeneration may be contributory to such biphasic changes [33]. Regarding the choroidal changes, early AMD seems to undergo biphasic alterations similarly; initially, choroidal volume and choroidal vascular index increased during early drusen formation but decreased thereafter. The authors think that such biphasic alterations suggest that changes in choroidal vascular anatomy may also drive and/or reflect the pathobiology of AMD progression [34].

However, different from eyes with SDDs, it remains unknown whether the function of the retina changes with an increased soft drusen load; a study demonstrated that, functionally, the amount of drusen within the central 3 mm of the macula does not appear to be related to low-luminance deficit in intermediate AMD [35]. 

The contribution of soft drusen to the pathogenesis of AMD, however, may be somewhat different between White and Asian populations. For example, in a multicenter prospective study that included 460 participants with neovascular AMD from Japan, only 131 eyes (28.5%) had hard drusen and 125 eyes (27.2%) had soft drusen in the macular area, supporting the notion that Japanese patients with neovascular AMD lack drusen [36]. 

#### 3.1.2. Refractile Drusen

**Figure 2:**
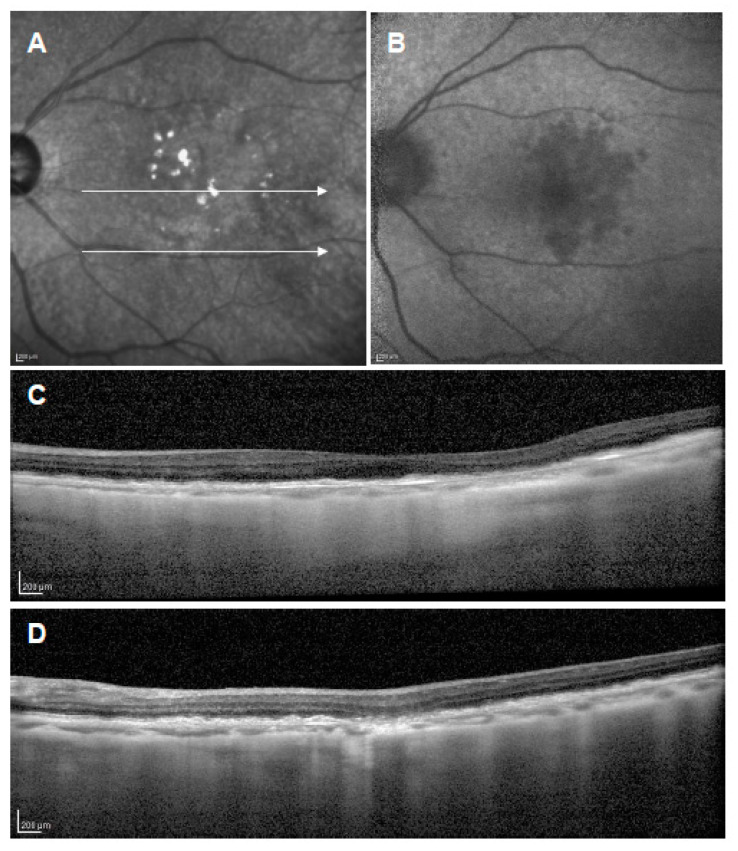
Refractile drusen. (**A**): Near-infrared reflectance image showing hyperreflectance lesions; (**B**): short-wavelength autofluorescence showing slightly decreased signal lesions; (**C**,**D**): corresponding OCT scans of white arrows in A showing laminar hyperreflectivity at the level of Bruch’s membrane and RPE atrophy.

Refractile drusen, or calcification of soft drusen, are defined as drusenoid material containing small refractile spherules (Figure 2). On OCT, calcification appears as heterogeneous internal reflectivity within drusen. Calcification of soft drusen occurs in a transition from intermediate AMD to GA. Less is known of their significance for MNV, although a study showed that the presence of refractile drusen at baseline may be a significant risk factor for RPE atrophy development after anti-vascular endothelial growth factor (VEGF) therapy for type 3 MNV [37]. AREDS demonstrated that the progression of GA was characterized by large drusen formation and hyperpigmentation, followed by regression of drusen and appearance of hypopigmentation; these are sometimes preceded by the appearance of refractile deposits [38], but regression of refractile drusen may also occur [39]. Other studies confirmed that refractile drusen are a relatively common feature observed in intermediate AMD and confer risk of the development of late AMD. Multimodal imaging study demonstrated that refractile drusen appear to be a stage of drusen regression marked by loss of RPE, thus contributing to the development of GA or cRORA [40].

In the histologic specimens, refractive drusen contained many small spherules rich in calcium phosphate. Ultrastructural study showed that these spherules are complex assemblies consisting of concentric shells containing thin layers of calcium. Such calcium-containing spherules appear to account for the glistening appearance. A recent study showed that these spherules are composed of hydroxyapatite and cause severe degeneration of the RPE [41].

#### 3.1.3. Subretinal Drusenoid Deposits

**Figure 3:**
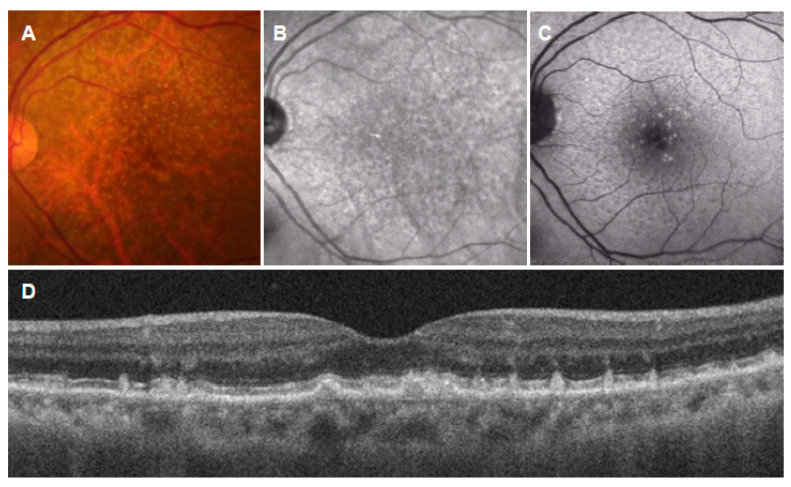
Subretinal drusenoid deposits (SDDs). (**A**): Color fundus photograph showing numerous drusen-like deposits; (**B**): near-infrared autofluorescence image showing dark spots with a bright central region; (**C**): short-wavelength autofluorescence image showing hyper- and hypoautofluorescent dots; (**D**): vertical OCT scan through the fovea showing drusenoid deposits in the subretinal space.

SDDs, also known as reticular pseudodrusen (RPD), confer a risk of GA (or cRORA) and MNV. On OCT, SDDs appear as distinct lesions that occur in the internal side of RPE that could extend internally through the EZ [42] (Figure 3). SDDs are commonly found in eyes with thinner choroids as yellowish-white net-like patterns on CFP and have a nodular or reticular morphology, namely, dot and ribbon SDD, and their identification often requires confirmations with OCT, fundus autofluorescence (FAF), and near infrared reflectance imaging. SDDs are more frequent in females, with increased age and more commonly being bilateral than unilateral. Although SDDs are relatively uncommon in some populations, such as Asians, eyes with SDDs have thinner choroids regardless of ethnicity [43,44]. A three-stage grading system based on OCT is also used for its classification [45].

Histological evaluation of SDDs revealed aggregations of material in the subretinal space between photoreceptors and RPE; they are granular extracellular deposits at the initial stage, and outer segment (OS) fragments and RPE organelles appear in some larger deposits. Degenerative features of photoreceptors in eyes with SDDs included OS disruption, intrusion into inner segments, and disturbance of neurosensory retina [46]. SDDs contain some proteins in common with soft drusen but differ in lipid composition. Some investigators assume that RPE-generated HDL may contribute lipid to SDDs [47].

SDDs are associated with an increased rate of progression of AMD, as shown in many phenotypical and functional studies. It is generally agreed that SDDs are a risk factor for the development of advanced AMD, for GA and MNV (type 3 neovascularization) [32]. SDDs are also associated with faster GA growth [48]. SDDs, like typical soft drusen, show highly dynamic changes [49], and regression of SDDs is associated with outer retinal atrophy, without necessarily causing RPE atrophy. Like GA growth, progression of outer retinal atrophy is faster in eyes with SDDs than in those without [50]. Interestingly, in some eyes with SDDs, the development of a vitelliform deposit, an entity not traditionally associated with AMD, was observed in a transition to advanced AMD [51].

Functionally, eyes with SDDs show slower dark adaptation (DA) and poorer contrast sensitivity when compared to eyes with typical soft drusen. Slow DA correlates with patient-reported functional deficits [52]. A prospective study enrolling 50 participants (11 controls, 17 intermediate AMD with no SDD, 11 intermediate AMD with SDD, and 11 non-foveal atrophic AMD) demonstrated that, compared with healthy participants, BCVA, LLVA, and scotopic thresholds were depressed and rod intercept time prolonged in intermediate AMD patients with SDD. Compared to eyes with intermediate AMD but without SDD, eyes with SDD also had reduced scotopic function. The function of eyes with SDD and non-foveal atrophy was not different, nor was that of healthy subjects compared with intermediate AMD without SDD. The study also demonstrated that BCVA, LLVA, and scotopic thresholds correlated well with outer nuclear layer (ONL) volume, ONL thickness, and choroidal thickness, supporting the notion that eyes with SDD are surrogate markers of photoreceptor abnormalities comparable with non-central atrophy [53]. Longitudinal decline in DA function, as measured by the rod intercept time prolongation, is accelerated in eyes with greater AMD severity and especially in eyes with SDD both at baseline and at 4 years’ follow-up [54].

SDDs are also found in other macular diseases, including pseudoxanthoma elasticum and acquired vitelliform lesions (AVLs). Their presence in AVLs, besides disrupted external limiting membrane and larger lesion area, is associated with an increased risk of macular atrophy progression [55]. 

#### 3.1.4. Pachydrusen

**Figure 4:**
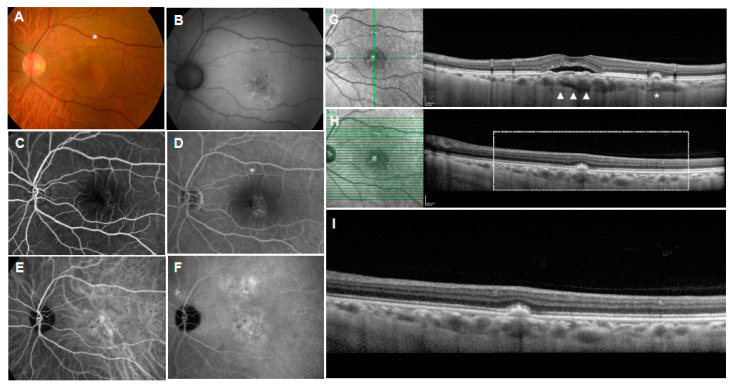
Multimodal imaging of pachydrusen in central serous chorioretinopathy (CSC). (**A**): Color fundus photograph showing pachydrusen (asterisk) located above the CSC; (**B**): near-infrared reflectance image showing dark center surrounded by bright regions in the site of pachydrusen; (**C**,**D**): fluorescein angiography showing late staining in the site of pachydrusen; (**E**,**F**): indocyanine green angiography showing enlarged choroidal vasculatures and choroidal hyperpermeability; (**G**): vertical OCT scan with corresponding infrared reflectance image showing the subretinal fluid, flat irregular pigment epithelial detachment (arrow heads), and pachydrusen (asterisk). Note the dilated choroidal vessels and thin choriocapillaris present beneath the corresponding site. (**H**): Horizontal OCT scan with corresponding infrared reflectance image showing pachydrusen (rectangle); (**I**): magnified OCT image of the rectangle.

Since the introduction of the concept that “disease expression in nonexudative AMD varies with choroidal thickness”, much attention has been paid to a new form of drusen, namely, pachydrusen [8] (Figure 4). Pachydrusen are typically observed under the RPE and are characterized by lesions which are larger and more distinct in shape and borders, with a more peripheral distribution, sparing the central macula compared to soft drusen. Pachydrusen have been recognized as a drusenoid deposit linked to pachychoroid, with characteristic findings such as a thickened choroid, dilation of Haller’s vessels with overlying attenuation of the choriocapillaris (CC), and hyperpermeability of the choroid. Indeed, Asian AMD patients are more likely to have pachydrusen and less likely to have pseudodrusen than White ethnic patients. Choroidal thickness is independently associated with drusen subtype, whereby thicker choroid is associated with pachydrusen and PCV, and thin choroid is associated with pseudodrusen [13]. In a Korean study, pachydrusen were prevalent in the typical neovascular AMD (40.4%) and PCV (47.8%) groups and were not detected in the retinal angiomatous proliferation group, and the prevalence of type 2 neovascularization was significantly lower in the pachydrusen group than in the subretinal drusenoid deposit group [44]. When various groups of AMD and “PSD” were classified into clusters according to the subfoveal choroidal thickness, eyes with pachydrusen were not clustered with eyes from other AMD groups; instead, they were classified in the same cluster as eyes from the PSD group [56]. Pachydrusen may be associated with several macular diseases other than MNV. Notably, pachychoroid is associated with central serous chorioretinopathy (CSC), a condition which is now included in PSD [57,58].

It is still controversial whether pachydrusen confer a high risk of developing advanced AMD. When the incidence of MNV was investigated in the fellow eye of unilateral neovascular AMD in a Korean population, the 5-year incidence of exudation was 20.9% among the 280 eligible eyes. However, in this study, pachydrusen did not confer a significantly increased risk compared to the no drusen group [59]. In a Singaporean study, which included a total of 75 eyes from 75 patients (29 with pachydrusen and 46 with soft drusen), there was no difference in the rate of progression to exudative MNV in the soft and pachydrusen groups (13.3% vs. 24.1%, *p* = 0.38). However, the authors also noted that pachydrusen, as compared to soft drusen, was associated with PCV subtype (85.7% vs. 16.7%, *p*  <  0.01), and the location of exudation was co-localized with soft drusen but not with pachydrusen [60]. In another Korean study, in eyes with pachydrusen, the 5-year incidence of neovascular AMD was 17.0%, and pachydrusen, in reference to soft drusen, was reportedly associated with the progression to PCV but not to typical neovascular AMD [61]. In contrast, in a Japanese hospital-based study, the 5-year incidence of neovascular AMD in the fellow eye was 32/296 (10.8%); however, no eyes with pachydrusen reportedly developed neovascular AMD [0/46 (0%)] [27]. In conclusion, when exudative changes due to MNV occur in the eyes with pachydrusen, they are more likely to be linked to pachychoroid-driven MNV, such as PCV and type I MNV, but whether pachydrusen confer a high risk of developing MNV is still unclear. 

In eyes with pachydrusen, focal disruptions of the ellipsoid zone/interdigitation zone (EZ/IZ) band can reportedly develop even without exudative change, possibly because of regression of drusenoid lesions. This may represent an atrophic form of pachychoroid manifestation [62]. Further studies are needed to confirm this finding.

Pachychoroid may be highly prevalent in Asian general populations, even though few studies have reported the prevalence of pachydrusen in a population-based cohort. A recent study showed that the age-adjusted prevalence of soft drusen, pachydrusen, and pseudodrusen was 4.3% (95%CI, 3.2–5.8%), 7.7% (95% CI, 6.2–9.7%), and 2.8% (95% CI, 1.7–4.2%), respectively, in a general Japanese population. It also showed that pachydrusen regress over time and may not be always associated with severe RPE atrophy as detected by CFP [63]. Interestingly, a population study demonstrated that choroidal thickness was associated with the pathology of intermediate AMD and its features in Asians; choroidal thickness was associated positively with the presence of intermediate AMD. In addition, among large drusen subtypes, choroidal thickness was associated positively with the presence of pachydrusen [12]. Some authors think that circulatory disturbances of the CC may contribute to the evolution of pachydrusen. For instance, indocyanine green angiography (ICGA) clearly demonstrates that pachydrusen in eyes with CSC frequently localized within the CC filling delay and corresponded to punctate hyperfluorescent spots on ICGA [58]. In support of this, another study investigated the relationship between pachydrusen and choroidal vascular hyperpermeability and punctate hyperfluorescent spots in patients with PCV or pachychoroid neovasculopathy and found significant correlations between punctate hyperfluorescent spots and pachydrusen. The authors considered that punctate hyperfluorescent spots may be a form of late staining “forme fruste” drusen, possibly associated with micro-ischemic changes to the CC [64]. 

#### 3.1.5. Cuticular Drusen

**Figure 5:**
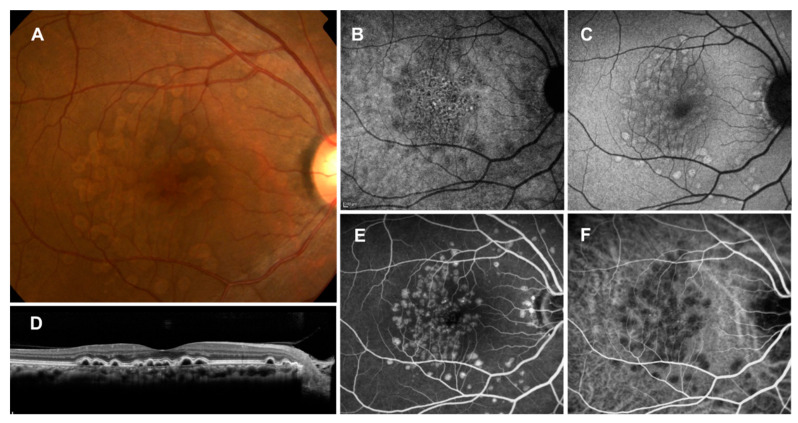
Cuticular drusen (large colloid drusen). (**A**): Color fundus photograph showing large yellowish drusen in the macula; (**B**): near-infrared autofluorescence showing hypoautofluorescent center surrounded by hyperautofluorescence; (**C**): short-wavelength autofluorescence showing mild hyperautofluorescence; (**D**): horizontal OCT scan through the fovea showing large colloid drusen under the RPE; (**E**): fluorescein angiography showing hyperfluorescence; (**F**): indocyanine green angiography showing hypofluorescence.

Cuticular drusen, also known as basal laminar drusen, may be associated with an increased risk of developing MNV or GA, especially when found in eyes of elderly patients (Figure 5). Cuticular drusen are characterized by a symmetrically distributed pattern in both eyes. On fluorescent angiography, they appear as numerous scattered, uniformly sized, small, hyperfluorescent drusen (starry-sky pattern) because of a central RPE atrophy from the triangular elevations of the RPE-basal lamina. Some drusen may be located outside the early treatment diabetic retinopathy study (ETDRS) standard 6 × 6 mm grid. Cuticular drusen can manifest at a relatively young age in the fifth decade of life, with a female preponderance [65]. Multimodal imaging using a combination of CFPs, OCT, fluorescein angiography, and FAF is essential for characterizing such lesions; as such, there are no population-based studies that investigated their prevalence in general populations. Histologically, small cuticular drusen appear as homogeneous and are similar to hard drusen. In larger cuticular drusen, fragmentation of the central and basal contents was frequently observed [66].

The diffuse involvement and/or accompanying large drusen in patients older than 60 years may confer a significant risk of either MNV or GA in White ethnic groups; macular complications of cuticular drusen were comparable with those of soft drusen in this age group [66,67]. The life cycles of cuticular drusen phenotypes were defined in a large cohort of White patients using multimodal imaging in a retrospective, observational cohort study involving 240 eyes of 120 clinic patients with a cuticular drusen phenotype in a White ethnic group, with a mean age of patients at the first visit of 57.9 ± 13.4 years. Drusen and RPE changes were seen in the peripheral retina, anterior to the vortex veins, in 21.8% of eyes. Of eyes with more than 5 years of follow-up, cuticular drusen disappeared in 58.3% of eyes; drusen coalescence was seen in 70.8% of eyes, and new RPE pigmentary changes developed in 56.2% of eyes. RPE abnormalities, AVLs, neovascularization, and GA occurred at a frequency of 47.5%, 24.2%, 12.5%, and 25%, respectively, and were significantly more common in patients older than 60 years of age. Such longitudinal studies from White ethnic populations clearly suggest that cuticular drusen confer a unique risk of the development of GA and MNV and are part of the overall spectrum of AMD [65,66]. There are, however, fewer reports from Asian countries. In a Korean study, large drusen, drusenoid pigment epithelial detachment (PED), GA, choroidal neovascularization (CNV), and AVL were observed in 59.3%, 26.5%, 18.5%, 3.7%, and 1.2% of eyes with cuticular drusen, respectively. The macular complication prevalence was reportedly similar between younger and older patients. Nonetheless, clinicians should use multimodal imaging to detect and be aware of the risk of progression to manifestations of GA and MNV. Although very rare, PCV may develop in eyes with cuticular drusen [68].

### 3.2. Pigment Abnormalities

Meanwhile, in the study of PCV evolution, it was found that pigment abnormalities alone (even when typical signs of drusen were absent) were an early sign of neovascular AMD in a Japanese population [14,15]. Another study from Japan also reported that large drusen as well as pigmentary abnormality may contribute to the development of neovascular AMD in the fellow eye of unilateral exudative AMD [27]. Interestingly, in a Japanese population study, choroidal thickness was found to be positively associated with the presence of non-AMD pigmentary abnormalities [12]. “Pigment abnormalities” is the term which was developed when CFP was the only available tool for the detection of the characteristic retinal features in AMD in epidemiological studies. Recent multimodal imaging studies, together with the novel understanding of the underlying cause of retina/RPE atrophy and MNV in AMD and PSD, have revealed several important prognostic features, especially OCT and OCTA biomarkers, associated with pigment abnormalities. 

#### 3.2.1. Intraretinal Hyperreflective Foci on OCT

**Figure 6:**
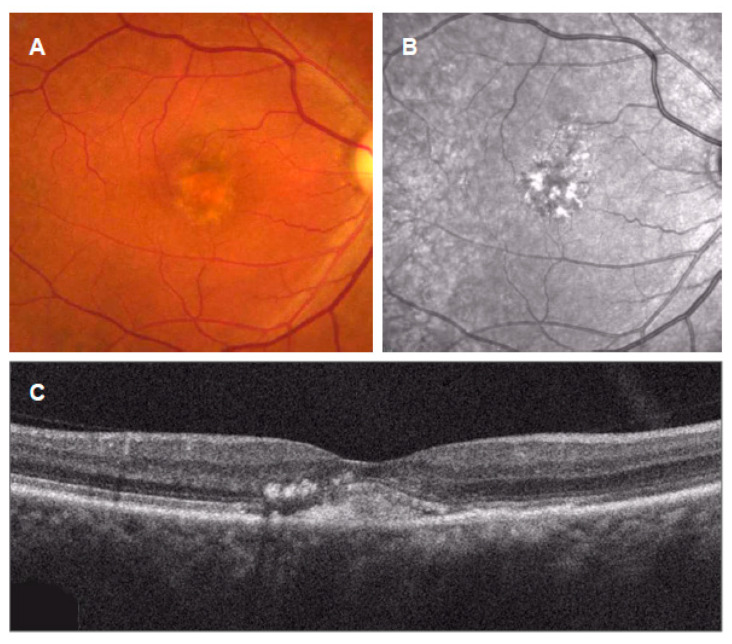
Intraretinal hyperreflective foci in acquired vitelliform lesions (AVLs). (**A**): AVLs appear in color fundus photograph; (**B**): near-infrared autofluorescence showing intensely hyperautofluorescent foci; (**C**): OCT scans showing subretinal location of vitelliform material with the intraretinal hyperreflective foci.

Retinal pigment abnormality, when observed on OCT, accompanies intraretinal hyperreflective foci, or small discrete hyperreflective lesions in the neurosensory retina (Figure 6). Intraretinal hyperreflective foci are a common occurrence in early-to-intermediate dry AMD. The area of intraretinal RPE migration on OCT correlates to areas of pigment clumping on fundus photography [69], and they are particularly useful for GA or cRORA risk evaluation [28].

There are numerous studies that suggest an association between intraretinal hyperreflective foci and progression of cRORA. Of note, a recent prospective study demonstrated that intraretinal hyperreflective foci had a largest effect size [HR of 5.21 (95% confidence interval [CI], 3.29–8.26)] among several OCT features associated with the progression from intermediate to late AMD, and the association remained significant when considering only the progression to cRORA and MNV alone, confirming the significance of intraretinal hyperreflective foci in the progression of cRORA and MNV [32].

Histological analysis suggests that intraretinal hyperreflective foci are the RPE cells, which are capable of hypertrophy and proliferation in response to CC ischemia; this is considered as one of the two main pathways of RPE fate: basolateral shedding of intracellular organelles (apparent apoptosis in situ) and activation with anterior migration. Migrated cells are packed with RPE organelles. RPE layer thickening due to cellular dysmorphia and thick basal laminar deposit is observed near the border of GA, suggesting that RPE activation and migration comprise an important precursor to atrophy [70].

#### 3.2.2. Pachychoroid Pigment Epitheliopathy

**Figure 7:**
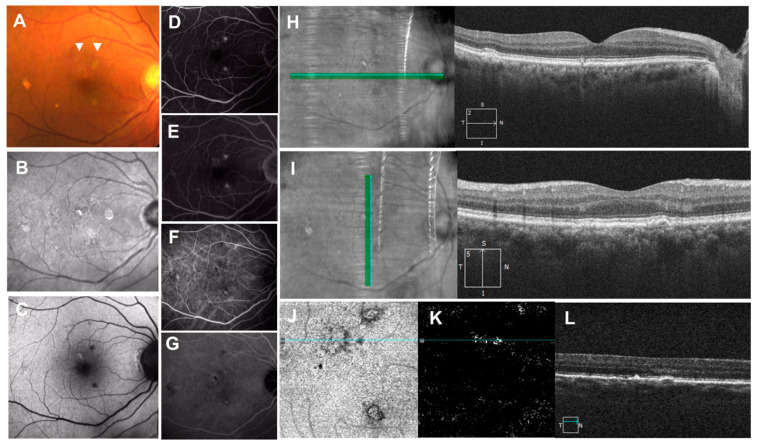
Pachychoroid pigment epitheliopathy (PPE). (**A**): Color fundus photograph showing “pigment abnormalities”, drusenoid and hyperpigmented lesions in the macula (arrow heads); (**B**): near-infrared autofluorescence showing hyperautofluorescence; (**C**): short-wavelength autofluorescence showing hyper- and hypoautofluorescence; (**D**): fluorescein angiography showing early staining with or without hypofluorescent center and (**E**): no late leakage; (**F**,**G**): indocyanine green angiography showing hypoautofluorescent center with or without surrounding hyperfluorescence; (**H**): horizontal OCT scan with the corresponding infrared image showing hyperreflective spike of RPE; (**I**): vertical OCT scan with corresponding infrared image showing RPE thickening and pigment epithelial detachment (PED); (**J**): en-face images from the OCT angiography demonstrating the slab extended from the RPE to RPE fit layers; (**K**): OCT angiography suggests presence of neovascularization in the layer; (**L**): associated cross-sectional OCT scan showing RPE thickening and shallow RPE elevation.

In the posterior pole over regions of choroidal thickening, retinal pigment abnormality may manifest as pachychoroid pigment epitheliopathy (PPE) (Figure 7). The areas of the pigment epitheliopathy are frequently at extrafoveal locations, but these lesions may be associated with the evolution of exudation and MNV involving the foveal center. PPE might have been misdiagnosed with pigmentary AMD and sometimes with pattern dystrophy or “retinal pigment epithelitis”. If pachychoroid features and RPE changes are observed in eyes with thickened choroid without subretinal fluid, the lesions are classified as having a PPE. FAF showed mottled hypoautofluorescence or hyperautofluorescent features. ICGA typically exhibited hyperpermeability overlapping with the area of the pigment epitheliopathy. PPE was considered as “forme fruste” CSC; it is frequently observed in uninvolved fellow eyes of patients with unilateral CSC and can ultimately progress to develop type 1 neovascularization (pachychoroid neovasculopathy; PNV) even in relatively young patients, with or without PCV, without necessarily developing CSC [4,71,72,73].

On cross-sectional OCT, PPEs are reported as foci of apparent RPE thickening or hyperplasia, serous or drusenoid PED, or double-layer sign (DLS). According to a recent study, RPE lesions can be further classified into microbreak, RPE thickening, hyperreflective spike of RPE, PED, and pachydrusen. Which feature(s) confer a high risk of developing exudative changes or atrophic changes are not still known, but, as mentioned in a following section, DLS may be associated with neovascularization [74]. 

Recent studies indicate that overlying neuroretinal atrophy may be accelerated in eyes with PPE. The ONL was found to be thinner in eyes with PPE even without exudation. As such, in PSD, the degenerative process may begin with RPE alterations before subretinal fluid accumulation. The authors suggest that PPE lesions, commonly seen above the pachyvessels, may be an indicator of photoreceptor apoptosis [75].

Some investigators think that PPE, CSC, PNV, and PCV can be now classified into four forms of a single disease entity, pachychoroid macular disease [76], although some differences exist between these eyes; for instance, the eyes of CSC patients had significantly smaller choroidal stromal areas than those of controls or of PPE, PNV, or myopic CNV patients [77].

#### 3.2.3. Double-Layer Sign on OCT

**Figure 8:**
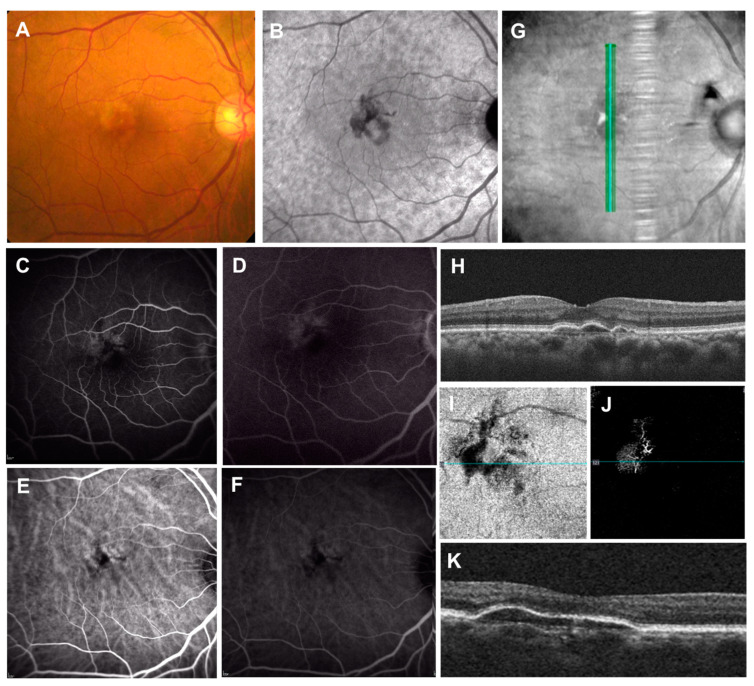
Double-layer sign (DLS) on OCT. (**A**): Color fundus photograph showing “pigment abnormalities” in the macula; (**B**): near-infrared autofluorescence showing mild hyperautofluorescent center surrounded by hypoautofluorescence; (**C**,**D**): fluorescein angiography showing parafoveal multifocal leakages; (**E**,**F**): indocyanine green angiography only showing enlarged choroidal vasculature; (**G**,**H**): vertical OCT scan with corresponding infrared reflectance image showing the DLS (shallow irregular retinal pigment epithelium elevation). Note the dilated choroidal vessels and thin choriocapillaris present beneath the corresponding site. (**I**,**J**): En-face images from the OCT angiography demonstrating the slab extended from the RPE to RPE fit layers. OCT angiography shows the presence of macular neovascularization. (**K**): Associated cross-sectional OCT scan showing the DLS.

OCT changes known as the DLS alert clinicians of the possible existence of nonexudative MNV (Figure 8). DLS may show minimal changes in CFP or may be associated with retinal pigment abnormalities (PPE), soft drusen, or drusenoid deposits (pachydrusen).

DLS was firstly described as a feature associated with the branching vascular network in PCV. It was described as “two highly reflective layers that consisted of RPE and another highly reflective layer beneath the RPE”. As such, they are also called shallow irregular RPE elevation (SIRE). The presence of a small dome-shaped PED with heterogeneous internal reflectivity and a connected DLS is highly suggestive of PCV [78]. However, DLS is not specific to PCV and can also be observed in eyes with other conditions, such as CSC, and, when found in eyes with exudative changes, DLS may be suggestive of MNV. In particular, hyperreflectivity and non-homogeneity of the sub-RPE space within DLS were independently associated with MNV in eyes with CSC [79]. Importantly, DLS is also found in eyes with nonexudative MNV, with or without drusen. These lesions may appear as a hot spot or plaque on ICGA, which had been considered as asymptomatic, subclinical nonexudative MNV in AMD eyes and had been shown to confer an increased risk of exudation. However, OCTA is more useful and is highly recommended to identify neovascular components in these nonexudative lesions.

### 3.3. Choroid and Choriocapillaris

Besides factors that are visible on CFP and OCT, there are several factors that can be visible only with advanced imaging tools. Among others, recent studies provided some evidence that the choroidal changes, including the CC changes, which can be visualized with the use of high-penetrate swept source OCTA, play important roles and are a potential risk factor for the development of late AMD including cRORA and MNV. Although imaging of CC has been a challenging field of research due to imaging artefacts, with recent refinements of the measurements, several investigations showed that CC flow deficits may be useful for enhancing risk stratification and prognostication of patients with intermediate AMD. For instance, CC flow deficit was significantly greater in intermediate AMD eyes that progressed to complete RORA [80]. Another study suggested that CC involvement may be somewhat different between cRORA and MNV development; CC flow deficits in the peripheral macula were reportedly associated with the development of cRORA, while CC FD in the center was associated with the development of MNV. Of note, CC flow deficit reportedly remained an independent risk factor when structural OCT biomarkers were considered [81]. Choroids are also affected in eyes with PSD. Reduced CC flow density, increased choroidal thickness, and CVH appear to co-localize in eyes with PPE [82]. Such flow disturbances may result in local ischemia, leading to the development of MNV or atrophy.

## 4. New AMD Terminology Based on Multimodal Imaging

With advances in imaging technology, mainly OCT and OCTA, the definitions of AMD and related terms have required revision. Although GA has been defined based on CFP or FAF images, OCT has recently enabled identification of atrophy in the early phase before detection by CFP or FAF as well as identification of the specific retinal and choroidal layers showing atrophy. The Classification of Atrophy Meeting (CAM) group proposed a consensus nomenclature and definition for atrophy based on OCT findings. They proposed four terms and showed their histological correlates: (i) cRORA, (ii) incomplete RPE and outer retinal atrophy (iRORA), (iii) complete outer retinal atrophy (cORA), and (iv) incomplete outer retinal atrophy (iORA). Notably, cRORA refers to macular atrophy with or without neovascularization, whereas GA represents cRORA but without neovascularization. The OCT criteria for the definition of cRORA are as follows: (1) a region of choroidal hyper-transmission of at least 250 μm in diameter; (2) a zone of attenuation or loss of the RPE of at least 250 μm in diameter; and (3) evidence of overlying photoreceptor degeneration. 

Similarly, OCT provides insights into the origins of neovascularization in both the retina and choroid. CNV has been used to describe neovascularization originating from choroid for decades, but neovascularization does not necessarily originate from the choroid [83,84,85,86]. The Consensus on Neovascular AMD Nomenclature (CONAN) group defined MNV as “an invasion by vascular and associated tissues into the outer retina, subretinal space, or sub-RPE space in varying combinations”, with three subtypes: type 1 MNV is an ingrowth of vessels initially from the CC into the sub-RPE space; type 2 MNV refers to the proliferation of new vessels arising from the choroid into the subretinal space; and type 3 MNV refers to a downgrowth of vessels from the retinal circulation toward the outer retina [87]. Notably, they do not directly correspond to the fluorescein angiographic categories of classic, occult, or mixed. 

OCTA technology has advanced and allows for the detection of flow signals in the sub-RPE basal lamina space [88], which has revealed the clinical importance of nonexudative type 1 MNV. Nonexudative MNV may have two phenotypes: one at risk of vision loss from exudation and one with preserved vision due to a surrogate CC [89]. The OCT signs suggestive of nonexudative MNV are SIRE [90] and DLS [91,92], which may appear as occult staining on FA or as a plaque of late hyperfluorescence in ICGA [93,94]. 

## 5. New Classifications Based on the Concept of AMD with Multimodal Imaging

**Figure 9:**
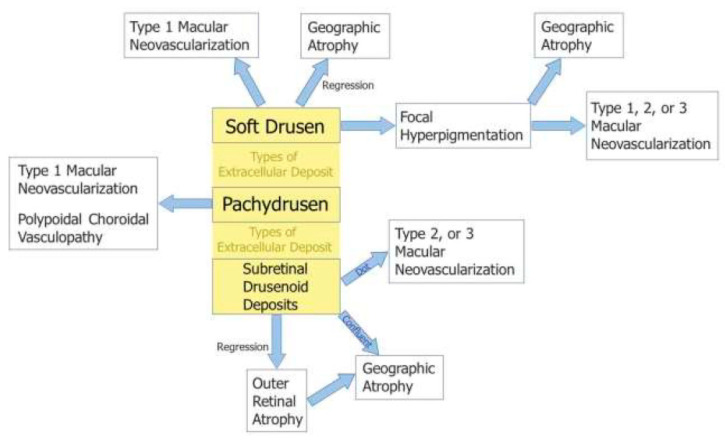
New classification system proposed by Spaide [5].

With advances in multimodal imaging technology, a few new classification systems have also been proposed based on revision of the disease concept of AMD. A new classification system [5] proposed by Spaide incorporates the following: (1) neovascularization subtypes; (2) deposit types (soft drusen, SDDs, and pachydrusen) and their features, such as choroidal thickness and topographical location; and (3) the association of specific deposit types with late AMD types (Figure 9). The classification encompasses the relevant disease presentations and can more precisely predict the clinical course related to specific deposits. 

**Figure 10:**
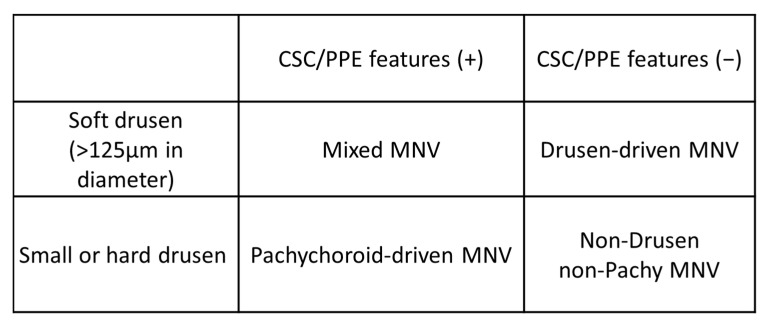
Classification of “typical neovascular age-related macular degeneration” proposed by Yanagi [11]. CSC: central serous chorioretinopathy; PPE: pachychoroid pigment epitheliopathy; MNV: macular neovascularization. “+”: present; ”−”:absent.

Drusen-dependent pathways and pachychoroid-driven pathways have recently been recognized as possible contributors to AMD pathology [10,11]. Building on this idea, Yanagi [11] proposed another classification and categorized MNVs into four groups based on the presence or absence of drusen and pachychoroid features (Figure 10): drusen-associated MNV is defined as the presence of drusen but without pachychoroid features; pachychoroid-driven MNV is defined as the presence of pachychoroid features but without drusen; mixed-type MNV is defined as the presence of both drusen and pachychoroid features; and idiopathic MNV is defined as the absence of both drusen and pachychoroid features. Given that pachychoroid-associated MNV does not necessarily depend on VEGF, the MNVs in distinct categories may respond differently to photodynamic therapy and anti-VEGF therapy [95,96,97]. Therefore, this classification could be useful for deciding the treatment strategy. 

## 6. Toward Refined AMD Classifications: A Proposal of a New Classification Incorporating Emerging Concept of Choroidal Pathology

Racial and ethnic differences in AMD have been recognized between Western and Asian populations. Polypoidal choroidal vasculopathy (PCV) is more common in Asians [26] than in Whites, and geographic atrophy is more frequent in Whites than in Asians [98]. Such differences are also seen in the early stage. Although the conventional classification system lacks the concept of choroidal pathology, a population-based study demonstrated that choroidal thickness is associated with the pathology of intermediate AMD in Asians [12]. Moreover, among large drusen subtypes, choroidal thickness was positively associated with the presence of pachydrusen [12]. These findings suggest the early involvement of the choroid in the pathogenesis of AMD, especially in Asians, and assessment of subtypes of deposits is necessary to evaluate AMD status. Thus, classifications incorporating choroidal pathology and subtypes of deposits are required in order to understand AMD.

In the new classification systems described above, Spaide incorporated the current understanding of the underlying causes of retinal/RPE atrophy and MNV in AMD and PSD, and Yanagi proposed another classification and categorized MNVs into four groups based on the presence or absence of drusen and pachychoroid features [11]. These two classifications draw on insights into disease pathology, including choroidal pathology, obtained from new imaging technologies. However, these classifications lack standardized definitions and diagnostic methods, especially for pachychoroid [99]. Further studies are warranted to verify these new classification systems based on multimodal analysis, which would expand them into severity scales with more precise predictive capabilities. 

**Figure 11:**
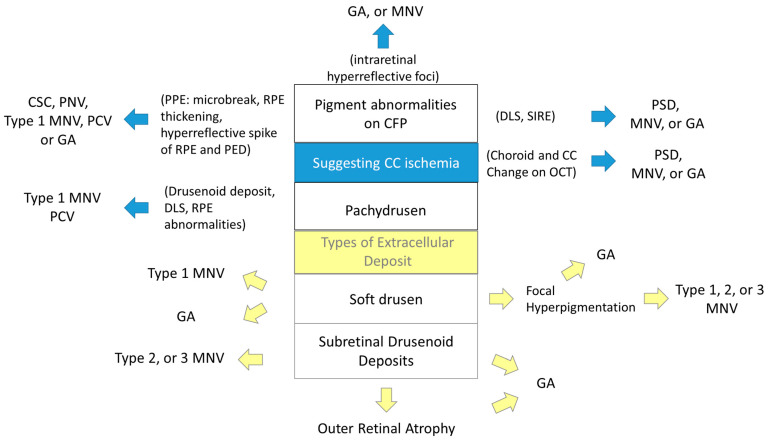
A proposal of a new classification incorporating choroidal pathology. GA: geographic atrophy; MNV: macular neovascularization; CSC: central serous chorioretinopathy; PNV: pachychoroid neovasculopathy; PCV: polypoidal choroidal vasculopathy; PPE: pachychoroid pigment epitheliopathy; RPE: retinal pigment epithelium; PED: pigment epithelial detachment; DLS: double-layer sign; SIRE: shallow irregular RPE elevation; PSD: pachychoroid spectrum disease.

Further, these classifications omit important prognostic features, namely, pigment abnormalities. For decades, pigmentary abnormalities have been considered a sign of early-stage AMD only when they coexist with at least medium drusen in the White population [16,17,18,19]. However, emerging evidence suggests that pigment abnormalities alone may contribute to the development of late AMD [14,15,25,27] and could be an independent sign of impending AMD (Figure 11). Although pigment abnormalities are a term used to describe CFP findings, they show various OCT signs: intraretinal hyperreflective foci, DLS (SIRE), and findings suggestive of PPE (microbreaks, RPE thickening, hyperreflective spikes of RPE and PED). These signs may arise from circulatory disturbances of the CC [74,82,100], which can result in local ischemia and the development of MNV or atrophy [80,81]. 

Moreover, choroidal thickness has been reported to be positively associated with the presence of non-AMD pigmentary abnormalities in Asians [12]. Therefore, we propose a new classification incorporating pigmentary abnormalities and their OCT signs as early signs of AMD or biomarkers of CC ischemia and pachychoroid, as with specific deposit types (Figure 11). The classification will be achieved by accumulation of evidence with detailed multimodal imaging technology, OCT and OCTA, with long-term follow-up studies with multiethnic participants. Notably, CC ischemia may have different effects on cRORA and MNV development.

## 7. Conclusions

With advances in multimodal imaging technology, the concept and classification of AMD have quickly evolved, but consensus is lacking on features associated with the progression of MNV. For example, the MNV in tilted disc syndrome is not classified as neovascular AMD, its pathology might, however, resemble that in pachychoroid. The same applies to PNV and PCV aged younger than 50 years. This review summarized new insights into MNV, focusing on early stages of AMD, and discussed early signs and their classifications. Additionally, the importance of pigmentary abnormalities as early signs of AMD or biomarkers of CC ischemia and pachychoroid was presented.

The choroid has been suggested to be deeply involved in the pathogenesis of AMD, and this is also true in the early stage. Therefore, the new classifications incorporating choroidal pathology are expected to be useful for understanding the pathology of AMD and determining the treatment strategy. Since the early stages of AMD are important preliminary steps in the disease, it is hoped that this article will contribute to preventing onset of the disease and slowing its progression.

## 8. Method of Literature Search

The authors conducted a search to capture references using the scholarly databases of PubMed published from inception to December 2021. The search involved MeSH headings and text terms. Explore with search terms including: “age-related macular degeneration”, “AMD”, “early AMD”, “intermediate AMD”, “late AMD”, “age-related maculopathy”, “ARM”, “macular neovascularization”, “MNV”, “early signs”, “drusen”, “drusen subtypes”, “pigmentary abnormalities”, “subretinal drusenoid deposit”, “SDD”, “reticular pseudodrusen”, “RPD”, “acquired vitelliform lesion”, “geographic atrophy”, “complete outer retinal atrophy”, “cRORA”, “outer retinal atrophy”, “cORA”, “pachychoroid”, “pachychoroid spectrum disease”, “polypoidal choroidal vasculopathy”, “PCV”, “pachychoroid pigment epitheliopathy”, “pachychoroid neovasculopathy”, “PNV”, “choriocapillaris”, “color fundus photography”, “imaging”, “multimodal imaging”, “optical coherence tomography”, “OCT”, “OCT angiography”, “OCTA”, “double layer sign”, “shallow irregular retinal pigment epithelium elevation”, “SIRE”, “epidemiology”, “prevalence”, “incidence”, “risk factors”, “associations”, ”classification”, “consensus”, “concept”, “terminology”, “Asian”. These terms were searched either independently or in conjunction with another term.

## Figures and Tables

**Table 1:** Summary of AMD classification systems.

	Definition of AMD Classifications				
AMD Stages	AMD Clinical Classification	International Classification	Original	AREDS Classification Simplified Severity * Scale	9-Step Severity Scale
**Normal eyes**	No drusen and no AMD pigmentary abnormalities	No signs of ARM or small/hard drusen (<63 µm) only	Category 1 No or few small drusen (<63 µm) and no RPE abnormalities Second eye: same as first eye	Score of 0 (plus fellow eye)	Step 1
**Normal aging**	Only drupelets (small drusen ≤ 63 µm) and no AMD pigmentary abnormalities				
**Early AMD**	Medium drusen (>63 µm and ≤125 µm) and no AMD pigmentary abnormalities	Soft drusen (≥63 μm) and RPE abnormalities	Category 2 Multiple small drusen (<20), a few intermediate drusen (≥63; <125 µm), or RPE abnormalities Second eye: same as first eye or category 1	Score of 0–1 (plus fellow eye)	Step 1–2
**Intermediate AMD**	Large drusen (>125 µm) and/or any AMD pigmentary abnormalities	N/A	Category 3 Extensive intermediate drusen, ≥ 1 large drusen (≥125 µm), or GA not involving center of fovea Second eye: same as first eye or category 1 or 2	Score of 1–2 (plus fellow eye)	Step 2–8
**Late/Advanced AMD**	nAMD and/or any GA	Late ARM is similar to AMD and includes dry AMD (GA in the absence of nAMD) or nAMD	Category 4 GA or nAMD Second eye: category 1, 2, or 3	N/A	Step 9

AMD: age-related macular degeneration; ARM: age-related maculopathy; RPE abnormalities: hyperpigmentation and/or hypopigmentation; AMD pigmentary abnormalities: any definite hyper- or hypopigmentary abnormalities associated with medium or large drusen but not associated with known disease entities; drupelets: small drusen (<63 μm); GA: geographic atrophy; nAMD: neovascular AMD; *: The AREDS simplified score assigns 1 point per eye for the presence of either one of the recognized risk factors (large drusen or pigment changes).

## Data Availability

Not applicable.

## References

[1] Lim L.S., Mitchell P., Seddon J.M., Holz F.G., Wong T.Y. (2012). Age-related macular degeneration. Lancet.

[2] Fleckenstein M., Keenan T.D.L., Guymer R.H., Chakravarthy U., Schmitz-Valckenberg S., Klaver C.C., Wong W.T., Chew E.Y. (2021). Age-related macular degeneration. Nat. Rev. Dis. Primers.

[3] Wong W.L., Su X., Li X., Cheung C.M., Klein R., Cheng C.Y., Wong T.Y. (2014). Global prevalence of age-related macular degeneration and disease burden projection for 2020 and 2040: A systematic review and meta-analysis. Lancet Glob. Health.

[4] Pang C.E., Freund K.B. (2015). Pachychoroid neovasculopathy. Retina.

[5] Spaide R.F. (2018). Improving the Age-Related Macular Degeneration Construct: A New Classification System. Retina.

[6] Warrow D.J., Hoang Q.V., Freund K.B. (2013). Pachychoroid pigment epitheliopathy. Retina.

[7] Spaide R.F., Gemmy Cheung C.M., Matsumoto H., Kishi S., Boon C.J.F., van Dijk E.H.C., Mauget-Faysse M., Behar-Cohen F., Hartnett M.E., Sivaprasad S. (2021). Venous overload choroidopathy: A hypothetical framework for central serous chorioretinopathy and allied disorders. Prog. Retina Eye Res..

[8] Spaide R.F. (2018). Disease Expression in Nonexudative Age-Related Macular Degeneration Varies with Choroidal Thickness. Retina.

[9] Sadda S.R., Guymer R., Holz F.G., Schmitz-Valckenberg S., Curcio C.A., Bird A.C., Blodi B.A., Bottoni F., Chakravarthy U., Chew E.Y. (2018). Consensus Definition for Atrophy Associated with Age-Related Macular Degeneration on OCT: Classification of Atrophy Report 3. Ophthalmology.

[10] Yanagi Y., Foo V.H.X., Yoshida A. (2019). Asian age-related macular degeneration: From basic science research perspective. Eye.

[11] Yanagi Y. (2020). Pachychoroid disease: A new perspective on exudative maculopathy. Jpn. J. Ophthalmol..

[12] Sasaki M., Ito Y., Yamasaki T., Yanagi Y., Gemmy Cheung C.M., Motomura K., Kawakami S., Kinoshita T., Yuki K., Hanyuda A. (2021). Association of Choroidal Thickness with Intermediate Age-Related Macular Degeneration in a Japanese Population. Ophthalmol. Retina.

[13] Cheung C.M.G., Gan A., Yanagi Y., Wong T.Y., Spaide R. (2018). Association between Choroidal Thickness and Drusen Subtypes in Age-Related Macular Degeneration. Ophthalmol. Retina.

[14] Sasaki M., Kawasaki R., Uchida A., Koto T., Shinoda H., Tsubota K., Wong T.Y., Ozawa Y. (2014). Early signs of exudative age-related macular degeneration in Asians. Optom. Vis. Sci..

[15] Ueta T., Iriyama A., Francis J., Takahashi H., Adachi T., Obata R., Inoue Y., Tamaki Y., Yanagi Y. (2008). Development of typical age-related macular degeneration and polypoidal choroidal vasculopathy in fellow eyes of Japanese patients with exudative age-related macular degeneration. Am. J. Ophthalmol..

[16] Klein R., Klein B.E., Tomany S.C., Meuer S.M., Huang G.H. (2002). Ten-year incidence and progression of age-related maculopathy: The Beaver Dam eye study. Ophthalmology.

[17] Wang J.J., Foran S., Smith W., Mitchell P. (2003). Risk of age-related macular degeneration in eyes with macular drusen or hyperpigmentation: The Blue Mountains Eye Study cohort. Arch. Ophthalmol..

[18] Mitchell P., Smith W., Attebo K., Wang J.J. (1995). Prevalence of age-related maculopathy in Australia. The Blue Mountains Eye Study. Ophthalmology.

[19] Klein R., Klein B.E., Linton K.L. (1992). Prevalence of age-related maculopathy. The Beaver Dam Eye Study. Ophthalmology.

[20] Klein R., Davis M.D., Magli Y.L., Segal P., Klein B.E., Hubbard L. (1991). The Wisconsin age-related maculopathy grading system. Ophthalmology.

[21] Bird A.C., Bressler N.M., Bressler S.B., Chisholm I.H., Coscas G., Davis M.D., de Jong P.T., Klaver C.C., Klein B.E., Klein R. (1995). An international classification and grading system for age-related maculopathy and age-related macular degeneration. Surv. Ophthalmol..

[22] Age-Related Eye Disease Study Research G. (2001). The age-related eye disease study (AREDS) system for classifying cataracts from photographs: AREDS report no. 4. Am. J. Ophthalmol..

[23] Ferris F.L., Davis M.D., Clemons T.E., Lee L.Y., Chew E.Y., Lindblad A.S., Milton R.C., Bressler S.B., Klein R., Age-Related Eye Disease Study Research G. (2005). A simplified severity scale for age-related macular degeneration: AREDS Report No. 18. Arch. Ophthalmol..

[24] Davis M.D., Gangnon R.E., Lee L.Y., Hubbard L.D., Klein B.E., Klein R., Ferris F.L., Bressler S.B., Milton R.C., Age-Related Eye Disease Study Group (2005). The Age-Related Eye Disease Study severity scale for age-related macular degeneration: AREDS Report No. 17. Arch. Ophthalmol..

[25] Ferris F.L., Wilkinson C.P., Bird A., Chakravarthy U., Chew E., Csaky K., Sadda S.R., Beckman Initiative for Macular Research Classification Committee (2013). Clinical classification of age-related macular degeneration. Ophthalmology.

[26] Maruko I., Iida T., Saito M., Nagayama D., Saito K. (2007). Clinical characteristics of exudative age-related macular degeneration in Japanese patients. Am. J. Ophthalmol..

[27] Notomi S., Shiose S., Ishikawa K., Fukuda Y., Kano K., Mori K., Wada I., Kaizu Y., Matsumoto H., Akiyama M. (2021). Drusen and pigment abnormality predict the development of neovascular age-related macular degeneration in Japanese patients. PLoS ONE.

[28] Balaratnasingam C., Messinger J.D., Sloan K.R., Yannuzzi L.A., Freund K.B., Curcio C.A. (2017). Histologic and Optical Coherence Tomographic Correlates in Drusenoid Pigment Epithelium Detachment in Age-Related Macular Degeneration. Ophthalmology.

[29] Jaffe G.J., Chakravarthy U., Freund K.B., Guymer R.H., Holz F.G., Liakopoulos S., Mones J.M., Rosenfeld P.J., Sadda S.R., Sarraf D. (2021). Imaging Features Associated with Progression to Geographic Atrophy in Age-Related Macular Degeneration: Classification of Atrophy Meeting Report 5. Ophthalmol. Retina.

[30] Klein R., Klein B.E.K., Linton K.L.P. (2020). Prevalence of Age-related Maculopathy: The Beaver Dam Eye Study. Ophthalmology.

[31] Curcio C.A. (2018). Soft Drusen in Age-Related Macular Degeneration: Biology and Targeting Via the Oil Spill Strategies. Investig. Ophthalmol. Vis. Sci..

[32] Nassisi M., Lei J., Abdelfattah N.S., Karamat A., Balasubramanian S., Fan W., Uji A., Marion K.M., Baker K., Huang X. (2019). OCT Risk Factors for Development of Late Age-Related Macular Degeneration in the Fellow Eyes of Patients Enrolled in the HARBOR Study. Ophthalmology.

[33] Chiang T.T., Keenan T.D., Agron E., Liao J., Klein B., Chew E.Y., Cukras C.A., Wong W.T. (2020). Macular Thickness in Intermediate Age-Related Macular Degeneration Is Influenced by Disease Severity and Subretinal Drusenoid Deposit Presence. Investig. Ophthalmol. Vis. Sci..

[34] Keenan T.D., Klein B., Agron E., Chew E.Y., Cukras C.A., Wong W.T. (2020). Choroidal Thickness and Vascularity Vary with Disease Severity and Subretinal Drusenoid Deposit Presence in Nonadvanced Age-Related Macular Degeneration. Retina.

[35] Ou W.C., Denlar R.A., Csaky K.G. (2020). The Relationship Between Central Drusen Volume and Low-Luminance Deficit in Age-Related Macular Degeneration. Transl. Vis. Sci. Technol..

[36] Tsujikawa A., Akagi-Kurashige Y., Yuzawa M., Ishibashi T., Nakanishi H., Nakatani E., Teramukai S., Fukushima M., Yoshimura N., AMD2000 Study Group (2018). Baseline data from a multicenter, 5-year, prospective cohort study of Japanese age-related macular degeneration: An AMD2000 report. Jpn. J. Ophthalmol..

[37] Hata M., Yamashiro K., Oishi A., Ooto S., Tamura H., Miyata M., Ueda-Arakawa N., Kuroda Y., Takahashi A., Tsujikawa A. (2017). Retinal Pigment Epithelial Atrophy after Anti-Vascular Endothelial Growth Factor Injections for Retinal Angiomatous Proliferation. Retina.

[38] Klein M.L., Ferris F.L., Armstrong J., Hwang T.S., Chew E.Y., Bressler S.B., Chandra S.R., AREDS Research Group (2008). Retinal precursors and the development of geographic atrophy in age-related macular degeneration. Ophthalmology.

[39] Oishi A., Thiele S., Nadal J., Oishi M., Fleckenstein M., Schmid M., Holz F.G., Schmitz-Valckenberg S. (2017). Prevalence, Natural Course, and Prognostic Role of Refractile Drusen in Age-Related Macular Degeneration. Investig. Ophthalmol. Vis. Sci..

[40] Suzuki M., Curcio C.A., Mullins R.F., Spaide R.F. (2015). REFRACTILE DRUSEN: Clinical Imaging and Candidate Histology. Retina.

[41] Tan A.C.S., Pilgrim M.G., Fearn S., Bertazzo S., Tsolaki E., Morrell A.P., Li M., Messinger J.D., Dolz-Marco R., Lei J. (2018). Calcified nodules in retinal drusen are associated with disease progression in age-related macular degeneration. Sci. Transl. Med..

[42] Spaide R.F., Ooto S., Curcio C.A. (2018). Subretinal drusenoid deposits AKA pseudodrusen. Surv. Ophthalmol..

[43] Nittala M.G., Song Y.E., Sardell R., Adams L.D., Pan S., Velaga S.B., Horst V., Dana D., Caywood L., Laux R. (2019). AMISH EYE STUDY: Baseline Spectral Domain Optical Coherence Tomography Characteristics of Age-Related Macular Degeneration. Retina.

[44] Lee J., Kim M., Lee C.S., Kim S.S., Koh H.J., Lee S.C., Byeon S.H. (2020). Drusen Subtypes and Choroidal Characteristics in Asian Eyes with Typical Neovascular Age-Related Macular Degeneration. Retina.

[45] Zweifel S.A., Spaide R.F., Curcio C.A., Malek G., Imamura Y. (2010). Reticular pseudodrusen are subretinal drusenoid deposits. Ophthalmology.

[46] Chen L., Messinger J.D., Zhang Y., Spaide R.F., Freund K.B., Curcio C.A. (2020). Subretinal Drusenoid Deposit in Age-Related Macular Degeneration: Histologic Insights Into Initiation, Progression to Atrophy, and Imaging. Retina.

[47] Lyssenko N.N., Haider N., Picataggi A., Cipollari E., Jiao W., Phillips M.C., Rader D.J., Chavali V.R.M. (2018). Directional ABCA1-mediated cholesterol efflux and apoB-lipoprotein secretion in the retinal pigment epithelium. J. Lipid Res..

[48] Bui P.T.A., Reiter G.S., Fabianska M., Waldstein S.M., Grechenig C., Bogunovic H., Arikan M., Schmidt-Erfurth U. (2022). Fundus autofluorescence and optical coherence tomography biomarkers associated with the progression of geographic atrophy secondary to age-related macular degeneration. Eye.

[49] Zhang Y., Wang X., Godara P., Zhang T., Clark M.E., Witherspoon C.D., Spaide R.F., Owsley C., Curcio C.A. (2018). Dynamism of Dot Subretinal Drusenoid Deposits in Age-Related Macular Degeneration Demonstrated with Adaptive Optics Imaging. Retina.

[50] Nittala M.G., Hogg R.E., Luo Y., Velaga S.B., Silva R., Alves D., Staurenghi G., Chakravarthy U., Sadda S.R. (2019). Changes in Retinal Layer Thickness in the Contralateral Eye of Patients with Unilateral Neovascular Age-Related Macular Degeneration. Ophthalmol. Retina.

[51] Spaide R.F., Yannuzzi L., Freund K.B., Mullins R., Stone E. (2019). EYES WITH SUBRETINAL DRUSENOID DEPOSITS AND NO DRUSEN: Progression of Macular Findings. Retina.

[52] Tan R., Guymer R.H., Luu C.D. (2018). Subretinal Drusenoid Deposits and the Loss of Rod Function in Intermediate Age-Related Macular Degeneration. Investig. Ophthalmol. Vis. Sci..

[53] Grewal M.K., Chandra S., Gurudas S., Rasheed R., Sen P., Menon D., Bird A., Jeffery G., Sivaprasad S. (2021). Functional clinical endpoints and their correlations in eyes with AMD with and without subretinal drusenoid deposits-a pilot study. Eye.

[54] Chen K.G., Alvarez J.A., Yazdanie M., Papudesu C., Wong W.T., Wiley H.E., Keenan T.D., Chew E.Y., Ferris F.L., Cukras C.A. (2019). Longitudinal Study of Dark Adaptation as a Functional Outcome Measure for Age-Related Macular Degeneration. Ophthalmology.

[55] Chandra S., Gurudas S., Narayan A., Sivaprasad S. (2021). Incidence and Risk Factors for Macular Atrophy in Acquired Vitelliform Lesions. Ophthalmol. Retina.

[56] Kim Y.H., Lee B., Kang E., Oh J. (2021). Clustering of eyes with age-related macular degeneration or pachychoroid spectrum diseases based on choroidal thickness profile. Sci. Rep..

[57] Takahashi A., Hosoda Y., Miyake M., Miyata M., Oishi A., Tamura H., Ooto S., Yamashiro K., Tabara Y., Matsuda F. (2021). Clinical and Genetic Characteristics of Pachydrusen in Eyes with Central Serous Chorioretinopathy and General Japanese Individuals. Ophthalmol. Retina.

[58] Matsumoto H., Mukai R., Morimoto M., Tokui S., Kishi S., Akiyama H. (2019). Clinical characteristics of pachydrusen in central serous chorioretinopathy. Graefes Arch. Clin. Exp. Ophthalmol..

[59] Lee J., Choi S., Lee C.S., Kim M., Kim S.S., Koh H.J., Lee S.C., Byeon S.H. (2019). Neovascularization in Fellow Eye of Unilateral Neovascular Age-related Macular Degeneration According to Different Drusen Types. Am. J. Ophthalmol..

[60] Teo K.Y.C., Cheong K.X., Ong R., Hamzah H., Yanagi Y., Wong T.Y., Chakravarthy U., Cheung C.M.G. (2021). Macular neovascularization in eyes with pachydrusen. Sci. Rep..

[61] Kim K.L., Joo K., Park S.J., Park K.H., Woo S.J. (2022). Progression from intermediate to neovascular age-related macular degeneration according to drusen subtypes: Bundang AMD cohort study report 3. Acta Ophthalmol..

[62] Lee J.H., Kim J.Y., Jung B.J., Lee W.K. (2019). Focal Disruptions in Ellipsoid Zone and Interdigitation Zone on Spectral-Domain Optical Coherence Tomography in Pachychoroid Pigment Epitheliopathy. Retina.

[63] Sato-Akushichi M., Kinouchi R., Ishiko S., Hanada K., Hayashi H., Mikami D., Ono S., Yanagi Y. (2021). Population-Based Prevalence and 5-Year Change of Soft Drusen, Pseudodrusen, and Pachydrusen in a Japanese Population. Ophthalmol. Sci..

[64] Kang H.G., Han J.Y., Kim M., Byeon S.H., Kim S.S., Koh H.J., Lee C.S. (2021). Pachydrusen, choroidal vascular hyperpermeability, and punctate hyperfluorescent spots. Graefes Arch. Clin. Exp. Ophthalmol..

[65] Sakurada Y., Parikh R., Gal-Or O., Balaratnasingam C., Leong B.C.S., Tanaka K., Cherepanoff S., Spaide R.F., Freund K.B., Yannuzzi L.A. (2020). CUTICULAR DRUSEN: Risk of Geographic Atrophy and Macular Neovascularization. Retina.

[66] Balaratnasingam C., Cherepanoff S., Dolz-Marco R., Killingsworth M., Chen F.K., Mendis R., Mrejen S., Too L.K., Gal-Or O., Curcio C.A. (2018). Cuticular Drusen: Clinical Phenotypes and Natural History Defined Using Multimodal Imaging. Ophthalmology.

[67] Fragiotta S., Fernandez-Avellaneda P., Breazzano M.P., Scuderi G. (2021). Clinical Manifestations of Cuticular Drusen: Current Perspectives. Clin. Ophthalmol..

[68] Fragiotta S., Kaden T.R., Freund K.B. (2018). Cuticular drusen associated with aneurysmal type 1 neovascularization (polypoidal choroidal vasculopathy). Int. J. Retina Vitreous.

[69] Ho J., Witkin A.J., Liu J., Chen Y., Fujimoto J.G., Schuman J.S., Duker J.S. (2011). Documentation of intraretinal retinal pigment epithelium migration via high-speed ultrahigh-resolution optical coherence tomography. Ophthalmology.

[70] Curcio C.A., Zanzottera E.C., Ach T., Balaratnasingam C., Freund K.B. (2017). Activated Retinal Pigment Epithelium, an Optical Coherence Tomography Biomarker for Progression in Age-Related Macular Degeneration. Investig. Ophthalmol. Vis. Sci..

[71] Dansingani K.K., Balaratnasingam C., Naysan J., Freund K.B. (2016). En Face Imaging of Pachychoroid Spectrum Disorders with Swept-Source Optical Coherence Tomography. Retina.

[72] Dansingani K.K., Balaratnasingam C., Klufas M.A., Sarraf D., Freund K.B. (2015). Optical Coherence Tomography Angiography of Shallow Irregular Pigment Epithelial Detachments In Pachychoroid Spectrum Disease. Am. J. Ophthalmol..

[73] Cheung C.M.G., Lee W.K., Koizumi H., Dansingani K., Lai T.Y.Y., Freund K.B. (2019). Pachychoroid disease. Eye.

[74] Karacorlu M., Ersoz M.G., Arf S., Hocaoglu M., Sayman Muslubas I. (2018). Long-term follow-up of pachychoroid pigment epitheliopathy and lesion characteristics. Graefes Arch. Clin. Exp. Ophthalmol..

[75] Ersoz M.G., Karacorlu M., Arf S., Hocaoglu M., Sayman Muslubas I. (2018). Outer Nuclear Layer Thinning in Pachychoroid Pigment Epitheliopathy. Retina.

[76] Abdin A.D., Suffo S., Fries F.N., Kaymak H., Seitz B. (2021). Uniform classification of the pachychoroid spectrum disorders. Ophthalmology.

[77] Lee M., Lee H., Kim H.C., Chung H. (2018). Changes in Stromal and Luminal Areas of the Choroid in Pachychoroid Diseases: Insights Into the Pathophysiology of Pachychoroid Diseases. Investig. Ophthalmol. Vis. Sci..

[78] Wang Y., Bo Q., Jia H., Sun M., Yu Y., Huang P., Wang J., Xu N., Wang F., Wang H. (2021). Small dome-shaped pigment epithelium detachment in polypoidal choroidal vasculopathy: An under-recognized sign of polypoidal lesions on optical coherence tomography?. Eye.

[79] Hagag A.M., Rasheed R., Chandra S., Jeffery G., Sivaprasad S. (2021). The Diagnostic Accuracy of Double-Layer Sign in Detection of Macular Neovascularization Secondary to Central Serous Chorioretinopathy. Am. J. Ophthalmol..

[80] Corvi F., Tiosano L., Corradetti G., Nittala M.G., Lindenberg S., Alagorie A.R., McLaughlin J.A., Lee T.K., Sadda S.R. (2021). Choriocapillaris Flow Deficits as a Risk Factor for Progression of Age-Related Macular Degeneration. Retina.

[81] Corvi F., Corradetti G., Tiosano L., McLaughlin J.A., Lee T.K., Sadda S.R. (2021). Topography of choriocapillaris flow deficit predicts development of neovascularization or atrophy in age-related macular degeneration. Graefes Arch. Clin. Exp. Ophthalmol..

[82] Sakurada Y., Fragiotta S., Leong B.C.S., Parikh R., Hussnain S.A., Freund K.B. (2020). Relationship between Choroidal Vascular Hyperpermeability, Choriocapillaris Flow Density, and Choroidal Thickness in Eyes with Pachychoroid Pigment Epitheliopathy. Retina.

[83] Brown D.M., Michels M., Kaiser P.K., Heier J.S., Sy J.P., Ianchulev T., AREDS Research Group (2009). Ranibizumab versus verteporfin photodynamic therapy for neovascular age-related macular degeneration: Two-year results of the ANCHOR study. Ophthalmology.

[84] Rosenfeld P.J., Brown D.M., Heier J.S., Boyer D.S., Kaiser P.K., Chung C.Y., Kim R.Y., MARINA Study Group (2006). Ranibizumab for neovascular age-related macular degeneration. N. Engl. J. Med..

[85] Heier J.S., Brown D.M., Chong V., Korobelnik J.F., Kaiser P.K., Nguyen Q.D., Kirchhof B., Ho A., Ogura Y., Yancopoulos G.D. (2012). Intravitreal aflibercept (VEGF trap-eye) in wet age-related macular degeneration. Ophthalmology.

[86] Dugel P.U., Jaffe G.J., Sallstig P., Warburton J., Weichselberger A., Wieland M., Singerman L. (2017). Brolucizumab Versus Aflibercept in Participants with Neovascular Age-Related Macular Degeneration: A Randomized Trial. Ophthalmology.

[87] Spaide R.F., Jaffe G.J., Sarraf D., Freund K.B., Sadda S.R., Staurenghi G., Waheed N.K., Chakravarthy U., Rosenfeld P.J., Holz F.G. (2020). Consensus Nomenclature for Reporting Neovascular Age-Related Macular Degeneration Data: Consensus on Neovascular Age-Related Macular Degeneration Nomenclature Study Group. Ophthalmology.

[88] Dansingani K.K., Freund K.B. (2015). Optical Coherence Tomography Angiography Reveals Mature, Tangled Vascular Networks in Eyes With Neovascular Age-Related Macular Degeneration Showing Resistance to Geographic Atrophy. Ophthalmic Surg. Lasers Imaging Retina.

[89] Chen L., Messinger J.D., Sloan K.R., Swain T.A., Sugiura Y., Yannuzzi L.A., Curcio C.A., Freund K.B. (2020). Nonexudative Macular Neovascularization Supporting Outer Retina in Age-Related Macular Degeneration: A Clinicopathologic Correlation. Ophthalmology.

[90] Narita C., Wu Z., Rosenfeld P.J., Yang J., Lyu C., Caruso E., McGuinness M., Guymer R.H. (2020). Structural OCT Signs Suggestive of Subclinical Nonexudative Macular Neovascularization in Eyes with Large Drusen. Ophthalmology.

[91] Sato T., Kishi S., Watanabe G., Matsumoto H., Mukai R. (2007). Tomographic features of branching vascular networks in polypoidal choroidal vasculopathy. Retina.

[92] Shi Y., Motulsky E.H., Goldhardt R., Zohar Y., Thulliez M., Feuer W., Gregori G., Rosenfeld P.J. (2019). Predictive Value of the OCT Double-Layer Sign for Identifying Subclinical Neovascularization in Age-Related Macular Degeneration. Ophthalmol. Retina.

[93] Guyer D.R., Yannuzzi L.A., Slakter J.S., Sorenson J.A., Hanutsaha P., Spaide R.F., Schwartz S.G., Hirschfeld J.M., Orlock D.A. (1996). Classification of choroidal neovascularization by digital indocyanine green videoangiography. Ophthalmology.

[94] Chang T.S., Freund K.B., de la Cruz Z., Yannuzzi L.A., Green W.R. (1994). Clinicopathologic correlation of choroidal neovascularization demonstrated by indocyanine green angiography in a patient with retention of good vision for almost four years. Retina.

[95] Chang Y.C., Cheng C.K. (2020). Difference between Pachychoroid and Nonpachychoroid Polypoidal Choroidal Vasculopathy and Their Response to Anti-Vascular Endothelial Growth Factor Therapy. Retina.

[96] Baek J., Lee J.H., Jeon S., Lee W.K. (2019). Choroidal morphology and short-term outcomes of combination photodynamic therapy in polypoidal choroidal vasculopathy. Eye.

[97] Hata M., Tagawa M., Oishi A., Kawashima Y., Nakata I., Akagi-Kurashige Y., Yamashiro K., Ooto S., Tamura H., Miyata M. (2019). Efficacy of Photodynamic Therapy for Polypoidal Choroidal Vasculopathy Associated with and without Pachychoroid Phenotypes. Ophthalmol. Retina.

[98] Rim T.H., Kawasaki R., Tham Y.C., Kang S.W., Ruamviboonsuk P., Bikbov M.M., Miyake M., Hao J., Fletcher A., Sasaki M. (2020). Prevalence and Pattern of Geographic Atrophy in Asia: The Asian Eye Epidemiology Consortium. Ophthalmology.

[99] Spaide R.F. (2021). The Ambiguity of Pachychoroid. Retina.

[100] Tiosano L., Byon I., Alagorie A.R., Ji Y.S., Sadda S.R. (2020). Choriocapillaris flow deficit associated with intraretinal hyperreflective foci in intermediate age-related macular degeneration. Graefes Arch. Clin. Exp. Ophthalmol..

